# Pioglitazone inhibition of lipopolysaccharide-induced nitric oxide synthase is associated with altered activity of p38 MAP kinase and PI3K/Akt

**DOI:** 10.1186/1742-2094-5-4

**Published:** 2008-01-18

**Authors:** Bin Xing, Tao Xin, Randy Lee Hunter, Guoying Bing

**Affiliations:** 1 Department of Anatomy and Neurobiology, 310 Davis Mills Building, University of Kentucky, Chandler Medical Center, 800 Rose Street, Lexington, KY 40536-0298, USA; 2 Department of Neurosurgery, Shandong Provincial Hospital, Shandong University, Jinan, China

## Abstract

**Background:**

Previous studies have suggested that peroxisome proliferator activated receptor-gamma (PPAR-γ)-mediated neuroprotection involves inhibition of microglial activation and decreased expression and activity of inducible nitric oxide synthase (iNOS); however, the underlying molecular mechanisms have not yet been well established. In the present study we explored: (1) the effect of the PPAR-γ agonist pioglitazone on lipopolysaccharide (LPS)-induced iNOS activity and nitric oxide (NO) generation by microglia; (2) the differential role of p38 mitogen-activated protein kinase (p38 MAPK), c-Jun NH(2)-terminal kinase (JNK), and phosphoinositide 3-kinase (PI3K) on LPS-induced NO generation; and (3) the regulation of p38 MAPK, JNK, and PI3K by pioglitazone.

**Methods:**

Mesencephalic neuron-microglia mixed cultures, and microglia-enriched cultures were treated with pioglitazone and/or LPS. The protein levels of iNOS, p38 MAPK, JNK, PPAR-γ, PI3K, and protein kinase B (Akt) were measured by western blot. Different specific inhibitors of iNOS, p38MAPK, JNK, PI3K, and Akt were used in our experiment, and NO generation was measured using a nitrite oxide assay kit. Tyrosine hydroxylase (TH)-positive neurons were counted in mesencephalic neuron-microglia mixed cultures.

**Results:**

Our results showed that pioglitazone inhibits LPS-induced iNOS expression and NO generation, and inhibition of iNOS is sufficient to protect dopaminergic neurons against LPS insult. In addition, inhibition of p38 MAPK, but not JNK, prevented LPS-induced NO generation. Further, and of interest, pioglitazone inhibited LPS-induced phosphorylation of p38 MAPK. Wortmannin, a specific PI3K inhibitor, enhanced p38 MAPK phosphorylation upon LPS stimulation of microglia. Elevations of phosphorylated PPAR-γ, PI3K, and Akt levels were observed with pioglitazone treatment, and inhibition of PI3K activity enhanced LPS-induced NO production. Furthermore, wortmannin prevented the inhibitory effect of pioglitazone on the LPS-induced NO increase.

**Conclusion:**

We demonstrate that pioglitazone protects dopaminergic neurons against LPS insult at least via inhibiting iNOS expression and NO generation, which is potentially mediated via inhibition of p38 MAPK activity. In addition, the PI3K pathway actively participates in the negative regulation of LPS-induced NO production. Our findings suggest that PPAR-γ activation may involve differential regulation of p38 MAPK and of the PI3K/Akt pathway in the regulation of the inflammatory process.

## Background

In the central nervous system microglia play a major role in the inflammatory process, and numerous activated microglia surround dopaminergic neurons in the substantia nigra (SN) of Parkinson's disease (PD) brains [1]. Uncontrolled microglial activation may be directly toxic to neurons by releasing various substances such as nitric oxide (NO), prostaglandin E2, superoxide, and proinflammatory cytokines such as interleukin-1β (IL-β), tumor necrosis factor-alpha, and interleukin-6 [2-5]. These molecules can induce dopaminergic neuron death [6-8], and inhibition of microglial activation can protect dopaminergic neurons [8-10].

Although the mechanisms underlying the pathogenesis of PD are not completely understood, excessive oxidative stress is thought to play a critical role, and much attention has been placed on NO as a key factor. At physiological concentrations, NO is relatively nonreactive and most of its actions are related to neurotransmitter release, neurotransmitter reuptake, neurodevelopment, synaptic plasticity, and regulation of gene expression [11]. However, excessive production of NO can lead to neurotoxicity due to its conversion into a number of more reactive derivatives, collectively known as reactive nitrogen species. At high concentrations NO reacts directly with superoxide, with the fastest biochemical rate constant currently known, to produce peroxynitrite, a strong lipid-permeable oxidant that can oxidize proteins, lipids, RNA, and DNA. Peroxynitrite can inhibit mitochondria complex I, complex II, cytochrome oxidase (complex IV), and the ATP synthase [12-14] as well as increase mitochondrial proton permeability [14]. In addition, NO can induce reactive oxygen and reactive nitrogen species production from mitochondria [15], which may also induce mitochondrial permeability transition [16], resulting in cellular injury and ultimately cell death. In the case of PD as well as in PD animal models, it has been demonstrated that activated microglia exhibit a robust expression of inducible nitric oxide synthase (iNOS) [3-5,17], and inhibition of iNOS provides neuroprotection to SN dopaminergic neurons against a variety of toxic insults [5,18-21].

Mitogen-activated protein kinases (MAPKs), including p38 MAPK, c-Jun NH(2)-terminal kinase (JNK), and extracellular signal-regulated protein kinase (ERK1/2), have been suggested to be involved in oxidative stress and proinflammatory signaling cascades, and evidence demonstrates that activation of p38 MAPK, JNK, and ERK1/2 signal cascades may be involved in lipopolysaccharide (LPS)-induced insults in microglia and cells derived from immortalized cell lines [20,22-25]. Activated microglia-induced neuronal death has been attributed to p38 MAPK and JNK activation [26], and a recent study showed that inhibition of JNK and p38-MAPK rescues dopaminergic neurons from a thrombin-activated microglia insult [27].

Nevertheless, the phosphoinositide 3-kinase (PI3K)/protein kinase B (Akt) pathway has been known to regulate cell growth, proliferation, glucose metabolism, transcription, protein synthesis, and cell survival [28]. In addition, PI3K/Akt regulates cellular activation, inflammatory responses, and apoptosis [29]. Recent studies have demonstrated that the PI3K/Akt pathway imposes a braking mechanism to limit the expression of proinflammatory mediators in LPS-treated monocytes by inhibiting the JNK and p38 MAPK pathways [30].

The peroxisome proliferator activated receptor-gamma (PPAR-γ) is a nuclear transcription factor reported in mammals in 1993 as an orphan receptor [31]. While it is mainly expressed in adipose tissue it also occurs in cells of the immune system, where it acts as a negative regulator of macrophage and microglia activation [32-34]. PPAR-γ forms a heterodimer with another nuclear receptor, retinoid X receptor alpha (RXRα). Upon activation of this complex, it binds to specific DNA sequence elements on target genes, termed peroxisome proliferator response elements, leading to responsive gene expression [35]. In addition, several studies have shown anti-inflammatory effects with PPAR-γ agonists. However, most of these effects are mediated via PPAR-γ independent mechanisms, including interference with nuclear factor-kappa B and activator protein-1 [36-42], phosphatase 2A [43], ERK [44], and JNK activity [45] via a process termed transrepression. For a more detailed review of PPAR-γ in inflammation see Daynes and Jones 2002 [46], and in microglia-mediated inflammation see Bernardo and Minghetti 2006 [47].

We previously showed that pioglitazone, a PPAR-γ agonist, provided neuroprotective properties to SN dopaminergic neurons in LPS-induced PD models both *in vivo *and *in vitro *[10,48], in which pioglitazone prevented LPS-induced expression of iNOS. In addition, we have demonstrated that pioglitazone may have therapeutic potential for the treatment of PD [10]. However, the potential differential regulation of iNOS expression and activity by p38 MAPK, JNK, and PI3K/Akt has not yet been explored. In the present study we examined the role of p38 MAPK, JNK, and PI3K/Akt in relation to the ability of pioglitazone to attenuate LPS-induced iNOS expression and NO production.

## Methods

### Animals

Timed-pregnant Sprague Dawley rats were obtained from Harlan (Indianapolis, IN, USA), and maintained in a pathogen-free environment. Housing, breeding, and experimental use of the animals were performed in strict accordance with the National Institutes of Heath guidelines and were approved by the Institute's Animal Care and Use Committee at the University of Kentucky.

### Reagents

Cell culture materials were obtained from Invitrogen (Carlsbad, CA, USA). Pioglitazone and *Salmonella minnesota *LPS was from Sigma-Aldrich (St Louis, MO, USA). The selective inhibitors were as follows: 1400W-iNOS inhibitor from Cayman Chemical (Ann Arbor, MI, USA), Cytosine β-D-arabinofuranoside hydrochloride from Sigma-Aldrich, SP600125-JNK inhibitor and SB203580-p38 inhibitor from A.G. scientific (San Diego, CA, USA), and wortmannin-PI3K inhibitor from Sigma-Aldrich. Antibodies used were: polyclonal anti-tyrosine hydroxylase (TH) antibody from Pel-Freez Biologicals (Rogers, AR, USA), polyclonal anti-iNOS from Millipore (Billerica, MA, USA), monoclonal anti-phospho p38 from Cell Signaling (Danvers, MA, USA), monoclonal anti-PPAR-γ (ser473) from Upstate (Billerica, MA, USA), polyclonal anti-PI3K p110 and polyclonal anti-Akt (Thr308) from Santa Cruz (Santa Cruz, CA, USA), and monoclonal anti-β-actin from Sigma-Aldrich (St Louis, MO, USA). The ABC kit and biotinylated secondary antibodies were purchased from Vector Laboratories (Burlingame, CA, USA).

### Mesencephalic neuron-microglia mixed cultures

Neuron-microglia mixed cultures were prepared from ventral mesencephalic tissues. Briefly, midbrain tissues were dissected from prenatal day 14 rat embryos in Ca^++^/Mg^++ ^free medium (CMF). Cells were dissociated via gentle mechanical trituration in Hanks' Balanced Salt Solution (HBSS) containing newborn calf serum (3.5:1 v/v), the concentration of the cell suspension was ~1.2 × 10^7 ^cells/ml before seeding, and the cells were seeded at 1 × 10^5 ^cells/well in poly D-lysine (50 μg/ml) pre-coated 24-well plates for immunocytochemistry, or at 2 × 10^6 ^cells/well in pre-coated 6-well plates for western blot. Cells were fed with minimium essential medium (MEM) containing 10% horse serum and 10% fetal bovine serum (FBS). Twenty-four hours later, 10 μM cytosine β-D-arabinofuranoside hydrochloride was added to suppress glial proliferation. Two to three days after seeding, the cells were replenished with 500 μl of fresh MEM with 5% horse serum and FBS. At DIV6 or DIV7 microglia (2 × 10^5 ^cells) were added to primary mesencephalic neuron-enriched cultures containing 1 × 10^5 ^cells per well and, after 24 hours, the cultures were treated with various protocols. DMSO was used as vehicle control since it was used to dissolve pioglitazone and other inhibitors.

### Microglia-enriched cultures

Primary glial cell cultures were established from the cerebral cortices of 2–3 day-old Sprague Dawley rat pups. Briefly, cerebral cortices were minced and gently dissociated by repeated pipeting in HBSS supplemented with newborn calf serum (3.5:1 v/v). Cells were collected by centrifugation (1000 g × 6 min), resuspended in dulbecco's modified eagle medium (DMEM/F-12) containing 10% FBS, penicillin (100 U/ml), and streptomycin (100 μg/ml), and were cultured on 175 cm^2 ^cell culture flasks in 5% CO_2 _at 37°C. Floating microglia were harvested at 2–8 weeks by shaking off at 200 rpm, where the final concentration of the cell suspension was ~1.4 × 10^6 ^microglial cells/ml. Microglia were re-seeded back into 24-well plates (2 × 10^5 ^cells) for NO assays and neuron-microglia mixed cultures. After 30 min, cultures were washed to remove non-adherent cells, and fresh medium was added. The purity of the microglial culture was >98% as determined by immunocytochemistry. Cultures were treated 24 hr after seeding the microglia.

### Immunocytochemistry

Culture medium was removed, the cells were rinsed in Tris buffer (pH = 7.3), fixed in 4% paraformaldehyde for 20 min, and rinsed again in Tris. Non-specific staining was blocked with 10% goat serum for one hour. Next, cells were incubated overnight at 4°C in primary TH antibody (1:10,000). After incubation in primary antibody, the cells were rinsed several times with Tris before a 1 hr incubation in the biotin-conjugated secondary antibody, goat anti-rabbit IgG (1:1000). This was followed by a series of rinses and incubation in the ABC-peroxidase reagent (Burlingame, CA. USA). The cells were rinsed and the color was developed with 3,3'-diaminobenzidine and 0.03% hydrogen peroxide in Tris buffer. Images were acquired using a Zeiss Axioplan 2 microscope connected to a digital Zeiss Axio camera operated by the AxioVision software. The TH-positive neurons were counted in each 24-well plate, and the percentage of control was reported. TH-immunostained neurons were considered healthy if they had at least two neurites and the length of all the neurites was two times longer than the diameter of the cell body.

### Nitrite oxide assay

The production of NO was assessed by the accumulation of nitrite in culture supernatants by using the colorimetric reaction of the Griess reagent. Culture supernatants were collected at different time points following LPS stimulation and were mixed with Griess reagent (0.1% N-[1-naphthyl] ethylenediamine dihydrochloride, 1% sulfanilamide, and 2.5% H_3_PO_4_). The absorbance at 548 nm was measured with a spectraMAX microplate reader from Molecular Devices (Sunnyvale, CA, USA).

### Western blot

Cells were collected and lysed for western blot. Protein concentrations were determined with the bicinchoninic acid assay following the manufacturer's guide. Equal amounts of protein were loaded, separated by PAGE gel electrophoresis, and were transferred to polyvinylidene difluoride membranes. Membranes were blocked with 5% nonfat milk and were incubated overnight at 4°C with polyclonal anti-iNOS antibody (1:1000), monoclonal anti-p38 (1:2000), monoclonal anti-PPAR-γ (1:250), polyclonal anti-PI3K p110 (1:250), polyclonal anti-Akt (1:250), or monoclonal anti-β-actin (1:4000). Peroxidase-linked anti-rabbit or anti-mouse IgG (1:4000) was used as the secondary antibody and the ECL Plus kit from Amersham Biosciences Inc (Piscataway, NJ, USA) was used for chemiluminecent detection. The optical density was measured using the scion image™ software (Frederick, MD, USA).

### Statistical analysis

The data are expressed as the means ± SEM and statistical significance was assessed by ANOVA followed by a Tukey comparisons test using the SYSTAT 10 software (SPSS Inc., Chicago, Illinois). A value of *p *< 0.05 was considered statistically significant.

## Results

### Pioglitazone inhibits LPS-induced nitric oxide generation in microglia-enriched cultures

**Figure 1:**
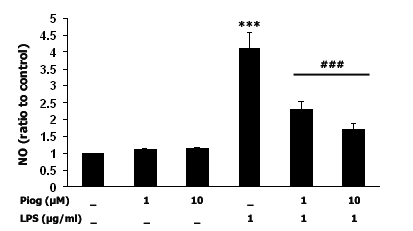
**Pioglitazone inhibits LPS-induced NO production in microglia-enriched cultures**. Microglia cultures were treated with pioglitazone (1 μM and 10 μM) 1 hr before LPS treatment, and 48 hrs later NO levels were measured. LPS significantly induced NO generation, and pretreatment with pioglitazone inhibited this LPS-induced NO production in a dose-dependent manner. Data presented are representative of three independent experiments (n = 3). (***p < 0.001 vs. control ###*p *< 0.001 vs. LPS)

To determine the effect of the PPAR-γ agonist pioglitazone on NO generation, two different doses of pioglitazone (1 μM and 10 μM) were administered to microglia-enriched cultures 1 hr before LPS (1 μg/ml) treatment. LPS induced a 4-fold increase in NO generation (*p *< 0.001) after 48 hr, and pretreatment with pioglitazone reduced NO production by about 40% to 60% (*p *< 0.001), respectively (Fig 1). Administration of pioglitazone concurrent with LPS, or 1 hr after LPS, failed to inhibit the LPS-induced NO increase (data not shown). In addition, pioglitazone alone did not alter NO production.

### Pioglitazone inhibits LPS-induced iNOS expression, and iNOS inhibition protects dopaminergic neurons from LPS insults in mesencephalic mixed cultures

**Figure 2:**
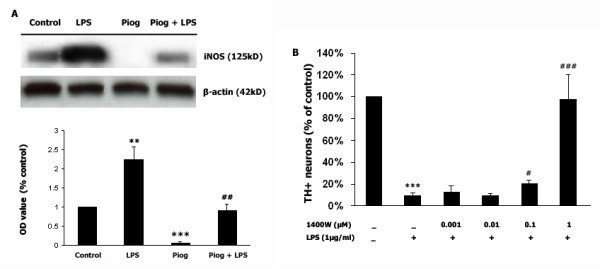
**Pioglitazone inhibits LPS-induced iNOS expression, and iNOS inhibition protects dopaminergic neurons from LPS insults**. Rat mesencephalic mixed cultures were treated with 1 μg/ml LPS for 48 hours. **A**: LPS treatment upregulated the expression of iNOS, and pretreatment with pioglitazone (10 μM), 1 hr before LPS, prevents its expression. **B**: Rat mesencephalic mixed cultures were treated with the selective iNOS inhibitor 1400 W, with different doses from 1 ng/ml to 10 μM/ml, 1 hr before a 72 hr LPS exposure. The number of TH-positive neurons was determined by immunocytochemistry. Data presented are representative of three independent experiments (n = 3). (**p < 0.01 vs. control, ***p < 0.001 vs. control, #*p *< 0.05 vs. LPS, ##*p *< 0.01 vs. LPS, ###*p *< 0.001 vs. LPS).

In this set of experiments, iNOS expression was determined by western blot performed 48 hrs after LPS (1 μg/ml) treatment. As shown in Fig 2A, basal iNOS expression was decreased by pioglitazone (*p *< 0.001), LPS treatment produced significantly enhanced iNOS expression (*p *< 0.01), and pretreatment with pioglitazone (10 μM) significantly reduced this LPS-induced increase in iNOS expression (*p *< 0.01). In addition, we used immunocytochemistry for TH-positive cells to assessed the effect of a specific iNOS inhibitor, 1400 W (1 nM to 10 μM), on the survival of dopaminergic neurons 72 hr after LPS treatment. Fig 2B shows that LPS induces a significant loss (90%) of the TH-positive neurons when the iNOS inhibitor is administered 1 hr before LPS (1 μg/ml). Partial neuroprotection against the LPS insult was seen when using 1400 W at 100 nM (*p *< 0.05) and 1 μM (*p *< 0.001).

### Pioglitazone reduces NO levels by inhibition of p38 MAPK activity

**Figure 3:**
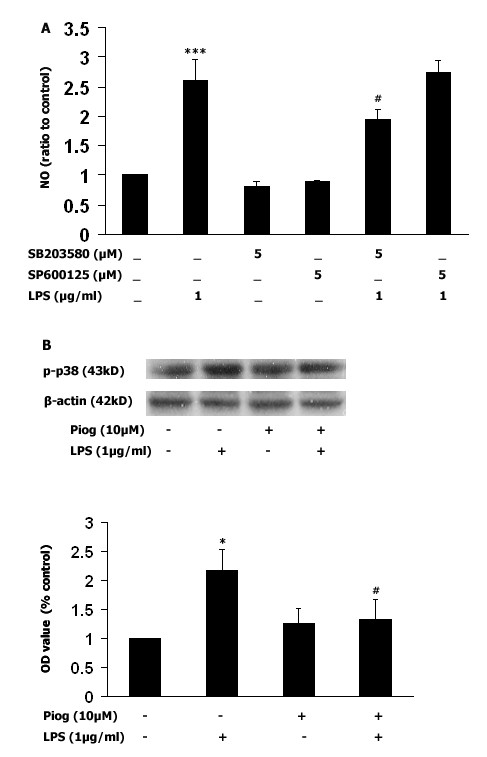
**Inhibition of NO by pioglitazone is related to inhibition of p38 MAPK activity**. **A**: A selective p38 MAPK inhibitor (SB203580), or a selective JNK inhibitor (SP600125), was added to microglia-enriched cultures 1 hr before LPS (1 μg/ml) exposure and, after 24 hrs, NO levels were measured. Only the p38 MAPK inhibitor prevented NO production. **B**: Pretreatment with pioglitazone inhibited LPS-induced phosphorylation of p38 MAPK in mesencephalic neuronal-microglia mixed cultures. Pioglitazone was added 1 hr before LPS treatment (1 μg/ml) and, after 30 mins, p38 MAPK was immunobloted. As shown in 3B, LPS increased phosphorylation of p38 MAPK, and pretreatment with pioglitazone inhibited this expression. Data presented are representative of three independent experiments (n = 3). (*p < 0.05 vs. control, ***p < 0.001 vs. control, #*p *< 0.05 vs. LPS).

**Figure 4:**
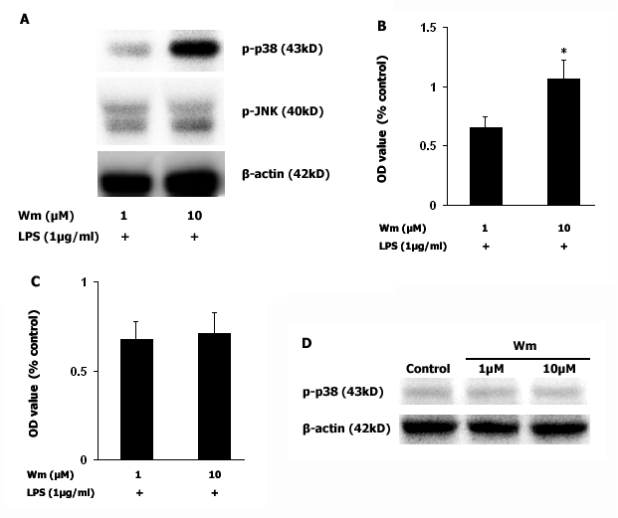
**Inhibition of PI3K activity increases LPS-induced p38 MAPK activity**. Wortmannin (1 μM and 10 μM) was administered to mesencephalic neuronal-microglia mixed cultures before LPS (1 μg/ml) was added and, after 30 mins, p38 MAPK was immunobloted. As shown in 4A, wortmannin enhances the phosphorylation of p38 MAPK under LPS stimulation in a dose-dependent manner (Fig 4A and 4B. *p *< 0.05), however, wortmannin did not increase p38 phosphorylation without LPS stimulation (Fig 4D). In contrast, inhibition of PI3K activity by wortmannin did not change JNK expression (Fig 4C). Data presented are representative of three independent experiments (n = 3). (*p < 0.05 vs. wortmannin 1 μM + LPS).

In the third part of our experiment, two proinflammatory pathways were examined, in order to demonstrate their involvement in the LPS-induced increase in NO production. Either SB203580 (a selective p38 MAPK inhibitor) or SP600125 (a selective JNK inhibitor) were administered to microglia-enriched cultures 1 hr before LPS (1 μg/ml) exposure. As shown in Fig 3A, LPS significantly increased NO generation (*p *< 0.001) and inhibition of p38 MAPK activity by pretreatment with SB203580 (5 μM) decreased this NO production (*p *< 0.05). Of particular interest, pretreatment with pioglitazone (10 μM) 1 hr before LPS (1 μg/ml) decreased phosphorylation of p38 MAPK (Fig 3B), and pretreatment with wortmannin (1 μM and 10 μM) increased LPS-induced p38 MAPK phosphorylation in a dose-dependent manner (Fig 4A and 4B. *p *< 0.05). An increase in phosphorylation of p38 MAPK was not found when wortmannin was administered alone, without LPS stimuli (Fig 4D). Wortmannin also did not change JNK expression (Fig 4A and 4C).

### Inhibition of PI3K activity prevents the inhibitory effect of pioglitazone on LPS-induced NO production

**Figure 5:**
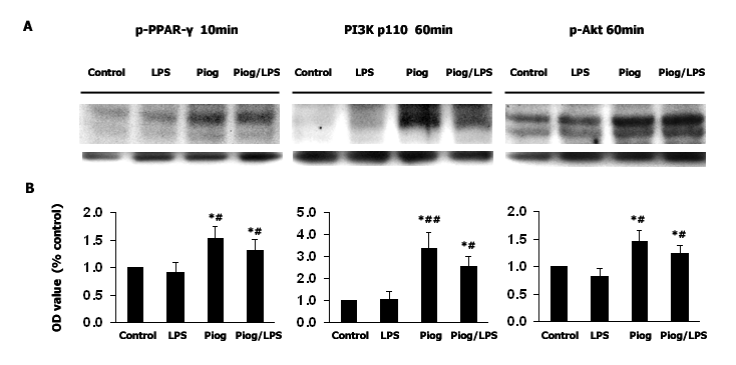
**Pioglitazone activates PPAR-γ and enhances PI3K/Akt activity**. Rat mesencephalic cultures (2 × 10^6 ^cells/well) were treated with pioglitazone (10 μM) only, or 1 hr before LPS (1 μg/ml) exposure. PPAR-γ activation was assessed after 10 min, and P13K and Akt were assessed after 60 min. PPAR-γ activation, PI3K and Akt expression were observed in the pioglitazone-treated cultures, compared to control and LPS-only groups. Data presented are representative of three independent experiments (n = 3). (*p < 0.05 vs. control, #*p *< 0.05 vs. LPS, ##*p *< 0.01 vs. LPS).

**Figure 6:**
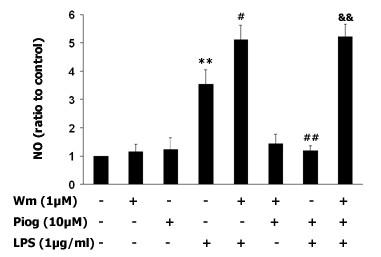
**PI3K negatively regulates the LPS-induced increase in NO production**. The specific PI3K inhibitor wortmannin (1 μM) was administered individually 90 mins before LPS treatment (1 μg/ml), or 30 mins before pioglitazone followed by LPS 60 mins later in microglia-enriched culture, and after 48 hrs NO levels were measured. The results show that the LPS-induced NO level was significantly higher than control (*p *< 0.01), and that pretreatment with pioglitazone inhibits LPS-induced NO (*p *< 0.01). In contrast, pretreatment with wortmannin enhanced the LPS-induced increase in NO generation (*p *< 0.05), and this pretreatment prevented the inhibitory effect of pioglitazone on LPS-induced NO generation. Data presented are representative of three independent experiments (n = 3).). (**p < 0.01 vs. control, #*p *< 0.05 vs. LPS, ##*p *< 0.01 vs. LPS, &&*p *< 0.01 vs. Piog plus LPS).

To determine if pioglitazone enhances PI3K/Akt expression and if its inhibition enhances LPS-induced NO generation, the levels of PI3K and Akt were determined. PPAR-γ, PI3K, and Akt phosphorylation were measured after LPS (1 μg/ml) exposure. As shown in Fig 5, PPAR-γ activation was observed in pioglitazone-treated cultures within 10 min after DMSO or LPS. PI3K and phosphorylated Akt were increased 60 min after LPS in the pioglitazone-treated cultures (Fig. 5, *p *< 0.05). Next, wortmannin (1 μM) was added 30 mins before pioglitazone (10 μM) treatment and the NO level was measured 48 h after LPS (1 μg/ml). The results showed that pretreatment with pioglitazone inhibited the LPS-induced NO increase (*p *< 0.01). However, when wortmannin was given 30 mins before pioglitazone, NO production was increased over LPS exposure (*p *< 0.05). Interestingly, administration of wortmannin (1 μM) 30 min before pioglitazone followed by LPS 1 hr later did not show the inhibitive effect of pioglitazone on NO level. Wortmannin alone, or together with pioglitazone, did not influence NO generation without LPS stimulation. Thus, pioglitazone prevents LPS-induced NO production, and pretreatment with wortmannin increases NO generation (Fig 6).

## Discussion

In our previous study, we reported that LPS injection into rat striatum induces a nigrostriatal inflammatory response, followed by dopaminergic neuronal loss, and that pioglitazone rescues dopaminergic neurons partially by inhibiting iNOS and COX-2 expression [10]. The present *in vitro *study was designed to investigate signal transduction pathways that may underlie the neuroprotection seen with pioglitazone against LPS exposure. We demonstrate that pioglitazone provides neuroprotective effects partially via reducing iNOS expression and NO generation from LPS-activated microglia. This appears to be associated with inhibition of p38 MAPK. In addition, pioglitazone increases PPAR-γ activation as well as PI3K/Akt activity, which may play a role in the inhibition of LPS-induced NO production.

### Pioglitazone inhibits LPS-induced iNOS and its inhibition protects dopaminergic neurons against LPS insult

Pretreatment of microglia-enriched cultures with pioglitazone (10 μM) significantly inhibited the LPS-induced increase in NO production (Fig 1). Previous studies have shown that pretreatment with pioglitazone decreases iNOS-positive cells in the SN and striatum of MPTP-treated mice [49] as well as decreases iNOS expression post intrastriatal LPS [10], intracerebellar LPS [50], and post *in vitro *LPS exposure [51], and these findings support our present results. In addition, we failed to observe any inhibitory effect of pioglitazone on LPS-induced NO production when pioglitazone was administered concurrent with LPS or 1 hr after LPS treatment, which suggests that PPAR-γ-mediated anti-inflammatory pathways and LPS-mediated inflammatory pathways might target and interact with common active molecules. There are several potential candidates that can be competitively targeted within these two pathways. The first candidate is LPS-induced MAPK activation. As Camp's study demonstrated using 293T cells, PPAR-γ can be phosphorylated by JNK and by p38 MAPK at its ser82 residue, and an increase in PPAR-γ phosphorylation may reduce its sensitivity to PPAR-γ ligands such as pioglitazone [52,53]. The second candidate is CD14, where LPS-induced microglia activation is mediated by CD14. However, the PPAR-γ agonist 15d-PGJ2 and rosiglitazone negatively regulate CD14 mRNA transcription in primary mouse microglia cultures [54]; although, a caveat to this finding is that 15d-PGJ2 was recently shown not to be a biologically relevant PPAR-γ agonist [38]. A third candidate for competitive targeting by LPS and PPAR-γ is RXR. Recent studies have shown that rosiglitazone inhibits LPS-mediated RXR nuclear export, resulting in increased nuclear binding of RXR in hepatocytes of mice [55], and that the RXR agonist, 9-cis retinoic acid, inhibits NO production by LPS-activated microglia [56]. In addition to the inhibition of LPS-induced NO production by pioglitazone, LPS-induced iNOS protein expression (as measured by immunoblotting) was prevented by pretreatment with pioglitazone (Fig. 2A). We previously demonstrated the ability of pioglitazone to attenuate the LPS-induced increases in iNOS expression [10]. We also observed some basal generation of NO, and almost no iNOS immunoreactivity in pioglitazone-treated cultures, suggesting that pioglitazone alone can inhibit iNOS expression. This basal NO may be generated by neuronal or endothelial NOS; however, we cannot rule out that the function of very limited iNOS is increased in a compensatory way, so that there is a basal generation of NO. Our results also demonstrate that inhibition of iNOS, with its specific inhibitor 1400 W, protects dopaminergic neurons against LPS-induced neurotoxicity. This data is supported by a previous study using iNOS inhibitors to attenuated dopaminergic neuron loss after intranigral LPS treatment [5]. Therefore, we speculate that pioglitazone protects dopaminergic neurons at least via inhibition of iNOS expression and function, which is consistent with other studies [10,21,49,57]. However, 1400 W, at 10 μM, did not protect TH-positive neurons (data not shown). Since 1400 W is a highly selective iNOS inhibitor that operates in a time-, dose-, and NADPH-dependent manner, it may bind iNOS to inhibit its function in the lower dose range [58,59] but, at higher concentrations, 1400 W might detach from iNOS leading to recovery of iNOS function. Another possibility is that iNOS and COX-2 cross talk with each other [60], that and once iNOS is inhibited, the function of COX-2 might be increased as a compensatory mechanism. Further work needs to be performed to determine this relationship.

### P38 MAPK is associated with LPS-induced NO generation and PI3K/AKT mediated p38 MAPK activity upon LPS stimuli

To further clarify which proinflammatory pathways might be involved in mediating the inhibition of LPS-induced NO by pioglitazone, selective inhibitors for p38 MAPK (SB203580 5 μM) and for JNK (SP600125 5 μM) were administered before LPS stimulation. It is interesting that inhibition of LPS-induced NO production was only observed with administration of the p38 MAPK inhibitor, but not with the JNK inhibitor, in microglia-enriched cultures. These results suggest that p38 MAPK might be associated with LPS-mediated iNOS regulation, but not with JNK. In addition, our study showed that pretreatment with pioglitazone before LPS (1 μg/ml) reduces phosphorylation of p38 MAPK (Fig 3B), which suggests that pioglitazone inhibits LPS-induced iNOS and NO production via suppression of p38 MAPK phosphorylation. Evidence has shown that inhibition of different MAPK pathways is associated with decreases in LPS-induced NO production [22], where the inhibitory effect of p38 MAPK has been more consistently observed [61,62]. In addition, our results are also consistent with two recent *in vivo *studies which suggest a role for p38 MAPK, but not JNK, in LPS-induced activation of iNOS [63,64].

Inhibition of PI3K with wortmannin did not enhance JNK phosphorylation upon LPS stimulation (Fig. 4A and 4C). In contrast, wortmannin enhanced p38 MAPK phosphorylation upon LPS stimulation in a dose-dependent manner (*p *< 0.05, Fig 4A and 4B), suggesting that PI3K/Akt mediated LPS-induced p38 MAPK activity and pioglitazone might inhibit LPS-induced NO generation via regulation of PI3K/Akt activity.

### Pioglitazone may inhibit LPS-induced NO generation via activation of PI3K/Akt pathway

The western blot study on the relationship of PPAR-γ activation and PI3K/Akt activity upon LPS stimuli showed a great amount of PPAR-γ phosphorylation with pioglitazone alone and with pioglitazone plus LPS, 10 mins after DMSO or LPS exposure, when compared to the control group and LPS group. This was accompanied by the enhanced level of PI3K and Akt phosphorylation in the pioglitazone alone or pioglitazone plus LPS group after a 60 min DMSO or LPS exposure. These results suggest that activation of the PI3K/Akt pathway by pioglitazone might be via PPAR-γ activation. Whether the activation of PI3K/Akt by pioglitazone is PPAR-γ dependent or independent needs to be further clarified.

Our present study shows that inhibition of PI3K activity significantly enhances LPS-induced NO production (Fig. 6). Furthermore, pretreatment with wortmannin (1 μM) prevented the inhibitory effect of pioglitazone on the LPS-induced increase in NO production, suggesting that inhibition of NO by pioglitazone is PI3K-dependent. Although several reports have demonstrated that LPS activates the PI3K pathway in mesangial cells, smooth muscle cells, and cell lines [65,66], studies on macrophages, whose morphology and phenotype are closer to those of microglia, show that inhibition of the P13K pathway enhances LPS-induced NO production [67]. Conversely, in the intrastriatal 6-OHDA PD model, transduction of neurons with the myristoylated form of Akt (Myr-Akt) has potent anti-apoptotic effects on dopaminergic neurons of the SN, sparing 80% of neuronal apoptosis. A more recent study demonstrated that human iNOS promoter induction by LPS/IFN-γ is suppressed by PI3K/Akt via inhibition of forkhead transcription factor FKHRL1 [68]. In addition, Akt can interact directly with mixed-lineage kinase 3, resulting in diminished JNK activation by mixed-lineage kinase 3. Kim et al demonstrated that Akt binds to apoptosis signal-regulating kinase 1, phosphorylates it at serine 83, and thereby reduces its kinase activity [69]. We did not find that LPS decreased PI3K or Akt levels as assessed by western blot, although there was a trend toward decreased PI3K and Akt phosphorylation in the pioglitazone plus LPS group, when compared to the pioglitazone alone group. This suggests that inhibition of LPS-induced NO generation by pioglitazone might occur independent of the LPS-induced inhibition of PI3K/Akt pathway; however, this needs further investigation. Although we observed that pioglitazone inhibited LPS-induced NO production via increasing PI3K/Akt activity and decreasing p38 MAPK phosphorylation, pioglitazone may also modulate NO production through other mechanisms. For instance, as a synthetic ligand for PPARγ, pioglitazone might inhibit iNOS, at least in part, through the repression of the activator of transcription 1 or nuclear factor-kappa B [70].

## Conclusion

Our present study shows that the PPAR-γ agonist, pioglitazone, significantly inhibits LPS-induced microglial increases in iNOS expression and NO production. This might be mediated by activation of the PI3K/Akt pathway, followed by inhibition of p38 MAPK activity, which may contribute to the inhibitory effects of pioglitazone on LPS-induced NO generation; thus, protecting dopaminergic neurons against LPS toxicity.

## List of abbreviations

Parkinson's disease (PD), peroxisome proliferator activated receptor-gamma (PPAR-γ), inducible nitric oxide synthase (iNOS), lipopolysaccharide (LPS), p38 mitogen-activated protein kinase (p38 MAPK), extracellular signal-regulated protein kinase (ERK1/2), c-Jun NH(2)-terminal kinase (JNK), phosphoinositide 3-kinase (PI3K), substantia nigra (SN), nitric oxide (NO), mitogen-activated protein kinases (MAPKs), retinoid X receptor α (RXR), tyrosine hydroxylase (TH), Ca++/Mg++ free medium (CMF), Hanks' Balanced Salt Solution (HBSS).

## Competing interests

The author(s) declare that they have no competing interests.

## Authors' contributions

Dr. Bing is the primary investigator (PI) in our lab. B Xing conceived the study and its design, performed the experiments, analyzed the data, and drafted the manuscript. T Xin and R Hunter took part in the western blot analysis and assisted in conceptual writing. All authors read and approved the final manuscript.
